# Clinical Performance of Short Expandable Dental Implants for Oral Rehabilitation in Highly Atrophic Alveolar Bone: 3-year Results of a Prospective Single-Center Cohort Study

**DOI:** 10.3390/medicina56070333

**Published:** 2020-07-03

**Authors:** Waldemar Reich, Ramona Schweyen, Jeremias Hey, Sven Otto, Alexander Walter Eckert

**Affiliations:** 1 Department of Oral and Plastic Maxillofacial Surgery, University Hospital Halle, Martin Luther University Halle-Wittenberg, Ernst-Grube-Straße 40, 06120 Halle (Saale), Germany; sven.otto@uk-halle.de (S.O.); alexander.eckert@uk-halle.de (A.W.E.); 2 Department of Prosthetic Dentistry, University School of Dental Medicine, Martin Luther University Halle-Wittenberg, Magdeburger Straße 16, 06108 Halle (Saale), Germany; ramona.schweyen@uk-halle.de (R.S.); jeremias.hey@uk-halle.de (J.H.)

**Keywords:** bone atrophy, expandable, oral health-related quality of life, short implant, stability

## Abstract

*Background and Objectives:* Oral health-related quality of life (OHRQOL) is compromised during the post-implant healing period, especially when vertical augmentation is required. A long-term trial sought to evaluate a short dental implant system with an apically expandable macro-design. *Materials and Methods:* Over 4.5 years, patients with limited vertical alveolar bone were consecutively recruited into this prospective cohort study. Implant success rate, OHRQOL (Oral Health Impact Profile (OHIP)-14), implant stability, and crestal bone changes were evaluated. *Results:* Data from 30 patients (mean age: 64.6 years, range 44–83) were analyzed, which related to 104 implants (53 in the maxilla, 51 in the mandible). Over the mean follow-up (42.6 ± 16.4 months), the implant success rate was 94.7% in the mandible (two implants lost) and 83.6% in the maxilla (four implants lost; *p* = 0.096), and the prosthetic success rate was 100%. The median OHIP-14 scores improved from 23 (interquartile range (IQR) 9–25.5) to 2 (IQR 0–5; *p* < 0.001). The mean implant stability quotient (ISQ) was 71.2 ± 10.6 for primary stability and 73.7 ± 13.3 (*p* = 0.213) for secondary stability, without significant maxilla-versus-mandible differences (*p* ≥ 0.066). Compared to the baseline, median crestal bone changes after loading were 1.0 mm (IQR 0–1.3) and 1.0 mm (IQR 0.2–1.2) in the maxilla and mandible (*p* = 0.508), respectively, at the end of the first year, 1.1 mm (IQR 0–1.3) and 1.0 mm (IQR 0.1–1.2) (*p* = 0.382), respectively, at the end of the second year, and 1.2 mm (IQR 0–1.9) and 1.1 mm (IQR 0.1–1.2) (*p* = 0.304), respectively, at the end of the third year. *Conclusions:* In patients with limited vertical bone height, short implants with optimized macro-design constitute a reliable method for functional rehabilitation, avoiding extensive alveolar bone augmentation.

## 1. Introduction

The treatment process is extensive for patients with limited vertical alveolar bone height because augmentation procedures are required prior to implanting standard dental implants [1]. However, the remodeling of augmented alveolar ridges occurs with volume loss, due to the remodeling or uncertain predictability of various developed procedures. Depending on general and local factors, up to 25% of the primary volume is resorbed [2]. For several years, short dental implants (<8 mm) have been an available, promising, and reliable treatment option for the orofacial rehabilitation of atrophic mandibles and maxillae, which constitutes an alternative to vertical ridge augmentation [3,4,5]. The prognosis of short dental implants and patient satisfaction with this treatment have been predictably high [6,7,8,9].

Biomechanical studies demonstrate that crestal bone is predominantly strained under axial and extra-axial loading [10]. However, the actual character of stress distribution in the peri-implant bone may vary according to anatomical and prosthetic aspects, as reported in some relevant studies [10,11,12,13,14,15,16,17]. Data suggest that a wide diameter should be preferred when using short implants. Finite element analyses have shown that peri-implant stress values and concentration areas decreased for cortical bone when the implant diameter was increased [12]. Moreover, increasing the implant diameter more effectively reduced crestal strain, compared to increasing the implant length (3.5-fold vs. 1.7-fold) [15].

Therefore, the macro- and micro-design of short dental implants should be optimized to improve their success rate and long-term stability (primary stability: immediately after implant insertion; secondary stability: after osseointegration; tertiary stability: under loading conditions). These innovations would benefit many people, including elderly patients with general comorbidities and co-medications [18]. As previously shown, oral health-related quality of life (OHRQOL) is compromised during the healing period following implant insertion [19], especially when augmentation procedures are required [8]. Yet, it is reasonable to reduce the overall treatment time for patients with atrophic alveolar ridges [20] by reducing invasiveness. Various groups of compromised patients (including those suffering from oral lichen planus and bone pathologies) could benefit from short implants allowing graftless rehabilitation and reduced invasiveness [21,22]. In terms of determining whether short dental implants could provide an alternative for the rehabilitation of cancer patients with deficient alveolar bone, survival rates between 75% and 96% (depending on local conditions) were found at 5 years [23].

Based on technical notes and initial experiences [24], this larger long-term trial sought to reevaluate a short dental implant system with an apically expandable macro-design. We hypothesized that the expandable macro-design of this short dental implant provides a reliable implant success rate, increases OHRQOL, ensures sufficient implant stability in a reduced vertical alveolar bone, and maintains peri-implant crestal bone.

## 2. Materials and Methods

### 2.1. Ethical Approval

All procedures in this study that involved human participants were performed in accordance with the ethical standards of the institutional research committee (date of issue: 21 August 2014, registration number *2014-60*) and with the 1964 Helsinki declaration and its later amendments.

### 2.2. Informed Consent

Informed consent was obtained from all individual participants that were included in the study.

### 2.3. Study Population and Measures

The participants in this study were recruited during implantological consultations at the Department of Oral and Plastic Maxillofacial Surgery, University Hospital Halle, Germany. The study was designed as a prospective, interventional, single-center, longitudinal cohort study that adhered to the STROBE criteria (see Supplementary Materials). The inclusion and exclusion criteria of adult patients interested in implantological treatment are outlined in Table 1. Patients were consecutively recruited over a 4.5-year period, beginning in the summer of 2014. Patients’ demographic data (age, gender), systemic condition, comorbidities and co-medications as well as dental status were recorded.

The measures employed were implant success and survival rate, as well as OHRQOL. The implant success rate was calculated using known success criteria (functional implant, no sign of infection or pain, no mobility, no radiolucent area around the implant) [25,26], while implant survival was computed using the Kaplan–Meier method. For implant survival analysis, only patients with a known observation time were included. Implant survival was defined as the “implant being osseointegrated and prosthetically in function” at the time of the latest follow-up examination. Oral Health Impact Profile 14 (OHIP-G14) questionnaires were evaluated at baseline (prior to implantation) and 6 months after prosthetic rehabilitation. Each OHIP item elicited information about how frequently subjects had experienced a specific impact in the previous month. OHIP-G14 is a self-administered questionnaire that follows a standard ordinal format (“*never*” = 0, “*hardly ever*” = 1, “*occasionally*” = 2, “*often*” = 3, and “*very often*” = 4). Seven dimensions of preoperative and postoperative OHIP-14 scores, as well as cumulative scores, were determined for each patient [27,28]. Oral health-related quality of life (OHRQOL) was re-assessed 6 months after prosthetic rehabilitation.

Implant stability and peri-implant crestal bone changes were also analyzed. Implant stability was measured by resonance frequency analysis (RFA) (Osstell AB, Göteborg, Sweden; Table 2) [29]. Implant stability quotient (ISQ) values were obtained using SmartPegs Types 17 and 35. According to each measurement in the vestibulo-oral direction, implant stability was classified as low with ISQ values <60, medium with ISQ values 60–70, and high with ISQ values >70 [30].

Crestal bone changes were evaluated yearly using digital periapical radiographs with the rectangular technique (see Section 2.6).

### 2.4. Implants

This study employed a CE-certified short expandable titanium dental implant (PYRAMIDION dental implant, DenTack Implants Ltd., Kfar-Saba, Israel). The implant was submitted to all mechanical fatigue tests in vitro (ISO 14801:2016 standard and FDA guidance for root-form endosseous dental implants and endosseous dental implant abutments). Apical expansion was performed after implant insertion using a special expansion tool and ratchet torque, which resulted in a pyramid shape [24]. The implants had the following dimensions and special characteristics: 5 mm, 6 mm, and 7 mm in length; 3.75 mm and 4.1 mm in diameter; internal (7 mm length) or external (5 mm and 6 mm length) hexagon platform.

Sample size calculation on the implant level was performed using the open-source statistical program G*Power 3.1, based on the *t*-test for differences between two independent means and considering the stability values in both jaws of the pilot study [31]. The total calculated sample size was 112 and the statistical power was 0.90.

### 2.5. Surgical and Prosthetic Protocol

Implantological treatment planning followed anamnesis, clinical, and orthopantomographic examination. Chair-side examination assessed cranial nerves, skin in the head and neck region, temporomandibular joints, masticatory muscles, and cervical lymph nodes. Intraoral examination included assessment of dental, periodontal, and mucosal pathologies, as well as edentulous alveolar ridges (Cawood and Howell category), occlusion, and existing restorations. Concerning the position and number of implants, the recommended categories from the German consensus conference were employed [32].

Prior to being enrolled in this study, all patients signed a written informed consent form. Surgical treatment was performed under local anesthesia. A mid-crestal incision was made and the mucoperiosteal flap was elevated (after a single median buccal release incision for edentulous jaws). Based on preoperative orthopantomography and intra-operative tactile sensation, the bone quality at the implant sites was recorded by the first author, using the classification from Lekholm and Zarb (D1, D2, D3, and D4) [33]. Table 2 summarizes the drilling sequence, manual implant insertion, and expansion. All surgeries, except for one patient (supervision by the first author), and all follow-up examinations were performed by the same surgeon (W.R.), so as to reduce performance and inter-observer variations/bias.

Implants were inserted 0.5 mm sub-crestally using a hand ratchet. Thereafter, the apical expansion was performed according to the manufacturer’s recommendations and using the appropriate expansion tool and hand ratchet. After a cover screw was positioned, a periosteal incision was made and mucosal wounds were closed using absorbable sutures (Monocryl 5-0, ETHICON, Johnson and Johnson, New Brunswick, NJ, USA). In some cases, lateral augmentation procedures were included, which were combined with antibiotic therapy consisting of amoxycillin 1 g every 8 h for 7 days. All patients were postoperatively instructed to use an oral antiseptic agent for 7 days (0.2% chlorhexidine) and to temporarily take the non-steroidal anti-inflammatory drug ibuprofen (600 mg).

Participants were instructed not to wear their dentures for 1 week following surgery and to avoid brushing at the surgical site. Patients were followed up after 7 days. The conventional dentures were subsequently relined with a soft material (Visco gel, Densply, Salzburg, Austria). The following conventional periods of submerged healing were chosen for this study: 3 months in the mandible and 6 months in the maxilla. During re-entry surgery, a minimum of 2 mm keratinized peri-implant soft tissue mucosa was considered, where needed, using curvilinear crestal or palatal/lingual para-crestal oblique incisions [34].

All prosthetic treatments were provided by two experienced specialists (R.S. and J.H.) in the Department of Prosthetic Dentistry of a university school of dental medicine. Prosthetic treatment was initiated after a minimum of 2 weeks following surgical re-entry. This required three sessions for fixed dentures, four sessions for removable dentures with ball attachments, six sessions for combined fixed-removable dentures, and seven sessions for removable dentures with jaw bars [24]. The abutment screws were fixed with a torque of 15Ncm, according to the manufacturer’s recommendation. Wherever possible, adjacent implants were primarily splinted (crowns, bar) and extra-axial loading during dynamic occlusion was avoided. However, in some cases, eccentric group guidance was achieved. In order to reduce overloading in the peri-implant bone and implant-abutment connection, the occlusal surface was designed to be smaller [13,14,18,35,36]. The first follow-up was scheduled for a maximum of 4 weeks later. Further follow-ups were scheduled quarterly. Patients that had attended regular check-ups for correct maintenance of prostheses were evaluated to determine their need for relining and changes to retention inserts.

### 2.6. Clinical and Radiological Follow-up

The first follow-up investigation was arranged for a maximum of 4 weeks after prosthetic rehabilitation. Further aftercare was arranged quarterly in the first year, and every 6 months thereafter. Patients were screened clinically and radiologically (yearly) for biological and technical complications. Marginal bone changes were evaluated yearly using digital periapical radiograms, ideally with the rectangular technique (Sidexis imaging software, Sirona, Bensheim, Germany). The distance between each implant shoulder and first bone-implant contact at the mesial and distal aspect was measured by the first author (W.R.), and the mean values per implant were calculated [37] 1, 2, and 3 years after loading. The known implant length served as a reference to verify the precise measurement of crestal bone changes. The authors applied Buser’s implant success criteria [25], which incorporate the absence of persistent subjective complaints, recurrent peri-implant infection, mobility, and continuous radiolucency, as well as the possibility of prosthetic loading. Crestal bone changes were classified according to Linkevicius (2019) as: “zero bone loss” = 0 mm, “stable bone remodeling” ≤ 1.2 mm, and “progressive bone loss” > 1.2 mm [38].

### 2.7. Data Gathering and Statistics

All patients were pseudonymized. Parameters were added to a databank and analyzed statistically using statistics software (IBM SPSS statistics, Version 20, Chicago, IL, USA). Descriptive statistics presented the distribution of several occurrences and frequencies, as well as combinations of certain features. The distribution of continuous data was tested by the Shapiro–Wilk test. Analytical statistical tests were performed depending on the scale, with a chi-squared test for categorical parameters, paired and independent *t*-tests for differences in mean values (normally distributed data), and a Wilcoxon signed rank test (for paired samples) or Wilcoxon rank sum test (for independent samples) for differences in median values (non-normally distributed data). The Bonferroni correction was used to counteract the problem of multiple comparisons. Implant survival was analyzed by Kaplan–Meier analysis and log-rank test. The level of significance was set at 5%.

## 3. Results

### 3.1. Study Population

Over a 43-month period (July 2014–January 2018), 34 patients (female n = 21, male n = 13) with an average age of 64.6 years (range 28–84 years) were enrolled in this study. The preoperative physical status classification, according to the American Society of Anesthesiologists, yielded: ASA Score 1 (healthy patient) in 15 patients, ASA Score 2 (patient with mild systemic disease: arterial hypertension, chronic bronchitis, diabetes mellitus, gastric ulcer, cured hepatitis C virus infection, hypothyroidism, osteoarthrosis, venous thrombosis) in 10 patients, and ASA Score 3 (patient with severe systemic disease: congestive heart failure, hemiplegia, history of a cured early stage oral squamous-cell carcinoma) in 5 patients. The anamnesis revealed that six patients were smokers. With regards to oral diseases, eight patients had a history of marginal periodontitis and two patients displayed chronic mucositis. Clinical examination demonstrated the following categories of edentulous alveolar process atrophy, according to Cawood and Howell: category III n = 29 and category IV n = 75. Indication categories related to the recommended implantological treatment [34] were distributed as follows: complete edentulous maxilla (IIIa) n = 5, complete edentulous mandible (IIIb) n = 9, partially edentulous areas confined to several teeth n = 2 (IIa) and n = 14 (IIb), and a partially edentulous area confined to a single tooth (Ib) n = 1.

**Figure 1:**
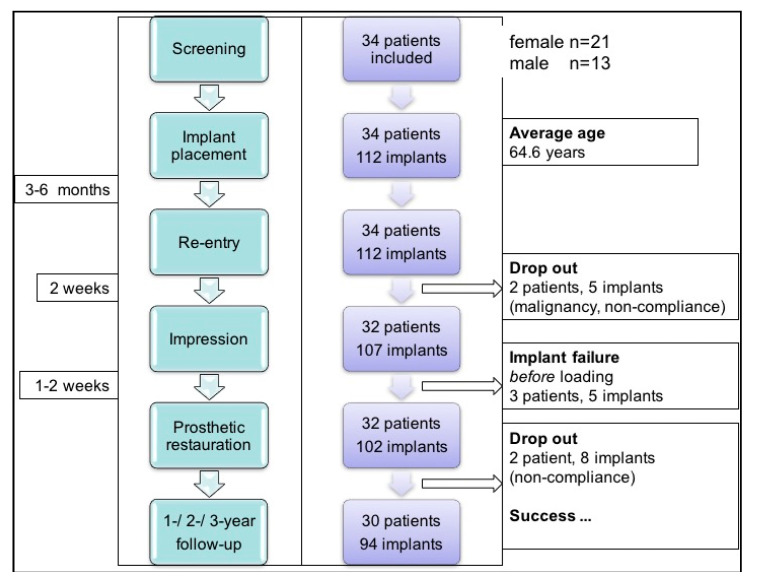
Study flowchart.

Figure 1 presents the flowchart for this study. In these patients, a total of 122 implants were inserted (maxilla n = 57, mandible n = 65). Over the total follow-up period, three patients dropped out due to non-compliance and one dropped out due to malignancy (total n = 13 implants).

Based on the radiological findings (preoperative orthopantomography) for 30 patients, the bone quality at implant sites was as follows: *D1* in n = 18 cases (maxilla n = 2, mandible n = 16), *D2* in n = 24 (maxilla n = 4, mandible n = 20), *D3* in n = 41 (maxilla n = 26, mandible n = 15), and *D4* in n = 21 (maxilla n = 21). The implant dimensions that were used were as follows: implant lengths of 5 mm in n = 7, 6 mm in n = 2, and 7 mm in n = 95; implant diameters of 3.75 mm in n = 29 and 4.1 mm in n = 75. Implant positions, prosthetic treatments, and success rates are summarized in Table 3. This table also contains nine patients (indicated by “#”) whose short-term results have previously been published [24]. In terms of validating the initial outcome, these individuals are included in the present analysis because of long-term follow-up. Four patients that dropped out are not included in Table 3.

Additional minor augmentation procedures were necessary in 12 patients at 23 implant sites. These included lateral augmentation with bone grafting (n = 4), bone spreading (n = 12), and internal sinus lift using an osteotome for condensing preparation after underdrilling and particulate bone grafting (n = 7). Patients were rehabilitated with fixed dentures in 11 cases and with removable dentures in 19.

**Figure 2:**
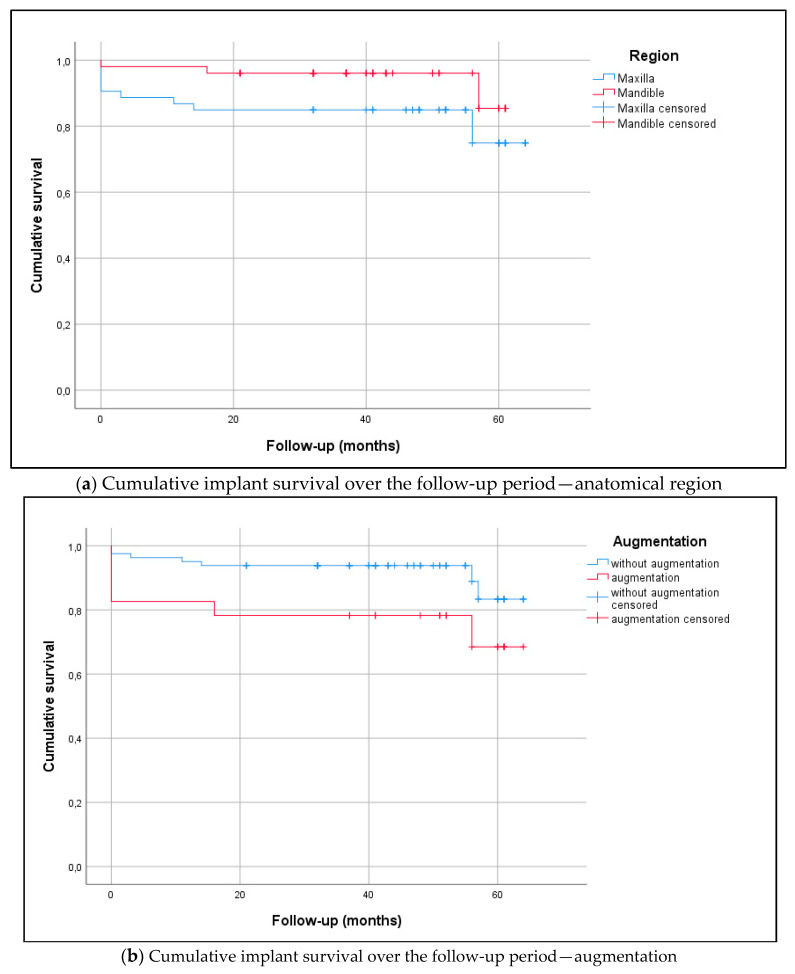
(**a**) The Kaplan–Meier diagram visualizes the analyses of implant survival in the maxilla (n = 51) and mandible (n = 51; log-rank test, *p* = 0.094). There were no statistically significant differences between the anterior and posterior region (log-rank test, *p* = 0.286) or in history of periodontitis (log-rank test, p = 0.465). The mean follow-up period was 42.6 ± 16.4 months (range 21–64) (Table 3). Overall survival rates in the maxilla and mandible were as follows: 1-year survival of 86.8% and 98.0%, respectively, 2-year survival of 84.9% and 96.1%, respectively, and 3-year survival of 84.9% and 96.1%, respectively. (**b**) The Kaplan–Meier diagram visualizes the analyses of implant survival relating to augmentation procedures. The overall survival rates returned statistically significant differences, as follows: 73.9% for implants with augmentation and 91.3% for implants without augmentation (log-rank test, p = 0.042). Implant sites with augmentation represented n = 23 (lateral augmentation n = 4, bone spreading n = 12, internal sinus lift n = 7) and implant sites without augmentation were n = 81.

The implant success rate over the mean follow-up period (42.6 ± 16.4 months; range 21–64 months) was 94.7% in the mandible (two implants lost) and 83.6% in the maxilla (four implants lost). Implant survival is presented in Figure 2 (Kaplan–Meier analysis, log-rank test, *p* = 0.096). In relation to systemic comorbidities (ASA score), the cumulative implant survival returned the following results: ASA Score 1 (healthy patient) 100%, ASA Score 2 (patient with mild systemic disease) 70.7%, and ASA Score 3 (patient with severe systemic disease) 95% (log-rank test, *p* < 0.001). Smoking was associated with poorer implant survival (76% vs. 91%; log-rank test, *p* = 0.017).

In relation to bone quality at implant sites (Lekholm and Zarb), the difference in cumulative implant survival was not statistically significant: D1 bone 83%, D2 bone 100%, D3 bone 83%, and D4 bone 86% (log-rank test, p = 0.275). Otherwise, the status of the alveolar process atrophy (Cawood and Howell) significantly influenced implant survival rate: category III 100% and category IV 83% (log-rank test, p = 0.011). This also significantly influenced prosthetic rehabilitation: removable 93% and fixed 100% (log-rank test, p < 0.001). In three patients, five implants were lost before loading, and four implants were lost under loading for a further three patients. The affected patients displayed compromised bone quality. This included tumor patients (squamous-cell carcinoma on the floor of the mouth), patients with highly atrophic alveolar bone (Cawood and Howell IV) [20], and patients with D3–D4 trabecular bone structures (according to Lekholm and Zarb) [33] (Table 3). In these patients, the prosthetic restauration was successfully modified. In one case (Patient 12), an abutment screw loosening was diagnosed and successfully refastened. No other technical complications were observed in terms of implant fracture, abutment screw fracture, or retention system fracture.

### 3.2. Oral Health-Related Quality of Life

Data were distributed non-normally for male and female patients’ preoperative (Shapiro–Wilk test, *p* ≤ 0.006) and postoperative OHRQOL (Shapiro–Wilk test, *p* ≤ 0.003). Table 4 presents seven dimensions of the OHIP-14 scores and related OHRQOL variables. Six months after completing rehabilitation (preoperative OHIP-14 vs. postoperative OHIP-14 Wilcoxon signed rank test for paired samples, *p* < 0.0001), OHIP-14 cumulative values yielded a significant change from male median 19 (IQR 2–24) and female median 23 (IQR 10–27; male vs. female Wilcoxon rank sum test for independent samples, *p* = 0.025) to male median 1 (IQR 0–2) and female median 3 (IQR 0.5–10; male vs. female Wilcoxon rank sum test for independent samples, *p* = 0.007). In relation to prosthetic status, calculation of OHIP-14 cumulative values revealed following significant differences: removable (baseline) median 24.5 (IQR 20–27) and fixed (baseline) median 10 (IQR 2–14; removable vs. fixed Wilcoxon rank sum test for independent samples, *p* < 0.0001); removable (rehabilitated) median 2 (IQR 1–10) and fixed (rehabilitated) median 1 (IQR 0–3; removable vs. fixed Wilcoxon rank sum test for independent samples, *p* = 0.030).

### 3.3. Biomechanical Implant Stability

**Figure 3:**
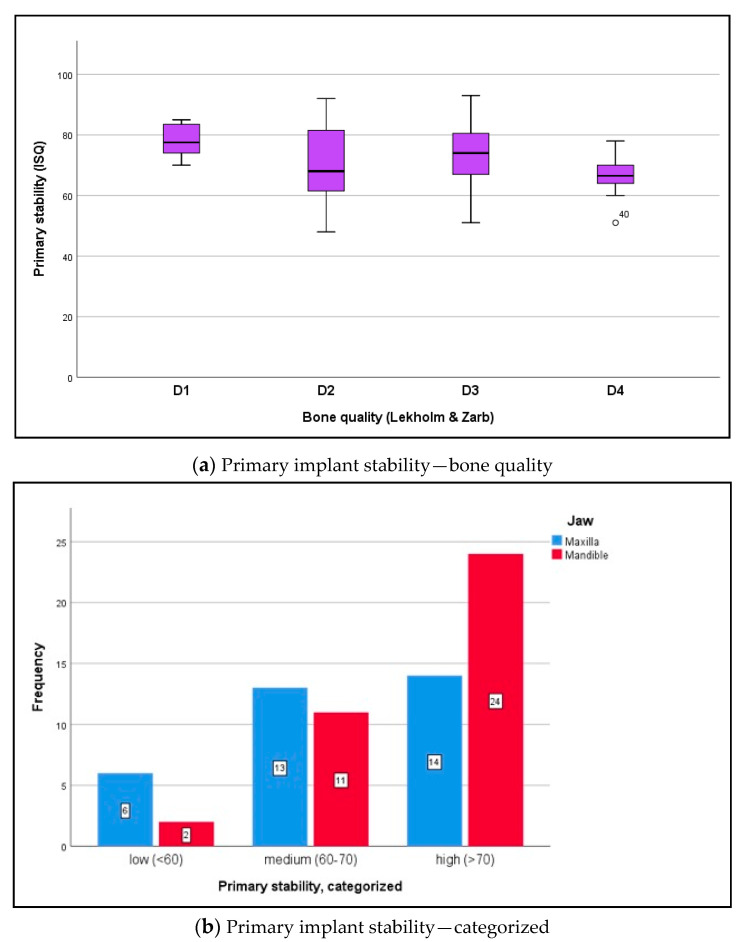
(**a**) The boxplot diagram visualizes the primary ISQ distribution for both jaws, which was measured intraoperatively using RFA (Osstell AB, Göteborg, Sweden): maxilla mean ISQ 66.1 ± 8.0, mandible mean ISQ 75.9 ± 10.6 (independent *t*-test, *p* = 0.099). In relation to bone quality at implant sites (Lekholm and Zarb), the following ISQ values were noted: *D1* bone 81.5 ± 5.0, *D2* bone 73.4 ± 11.5, *D3* bone 72.5 ± 10.6, and *D4* bone 63.1 ± 6.2. The differences in primary ISQ are *partially* statistically significant: *D1* versus *D2 p* *= 0.009*; *D2* versus *D3 p* = 0.420; *D3* versus *D4 p* = 0.294; independent *t*-test, Bonferroni correction. (**b**) According to the measurements (analyzable implants ∑ n = 70), implant stability was classified as low with ISQ values <60 (n = 8; 11.4%), medium with ISQ values 60–70 (n = 24; 34.3%), and high with ISQ values >70 (n = 38; 54.3%) [30]. The differences between the maxilla and mandible were not statistically significant (chi-squared test, *p* = 0.101).

**Figure 4:**
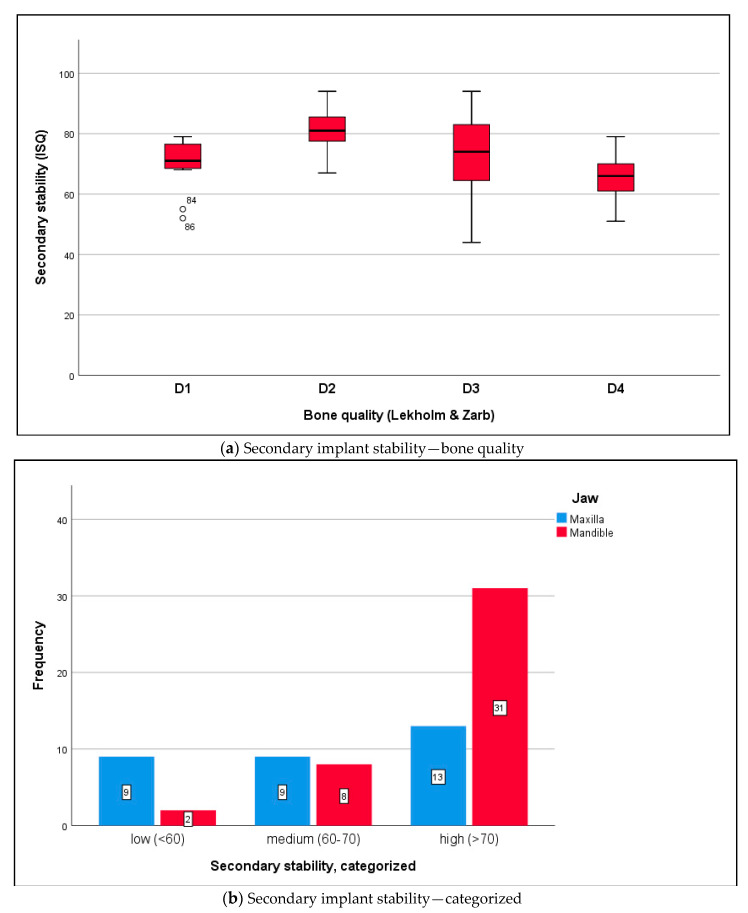
(**a**) The boxplot diagram shows the secondary ISQ distribution of osseointegrated implants: maxilla mean ISQ 68.2 ± 13.6, mandible mean ISQ 80.1 ± 9.3 (independent *t*-test, *p* = 0.174). In relation to bone quality at implant sites (Lekholm and Zarb), the following ISQ values were measured: *D1* bone 78.0 ± 1.4, *D2* bone 82.6 ± 8.4, *D3* bone 74.0 ± 13.7, and *D4* bone 66.3 ± 9.4. The differences in secondary ISQ were not statistically significant: *D1* versus *D2 p* = 0.862; *D2* versus *D3 p* = 0.180; *D3* versus *D4 p* = 0.081; independent *t*-test, Bonferroni correction. (**b**) According to the measurements (analyzable implants ∑ n = 72), implant stability was classified as low with ISQ values < 60 (n = 11; 15.3%), medium with ISQ values 60–70 (n = 17; 23.6%), and high with ISQ values >70 (n = 44; 61.1%) [30]. Differences between the maxilla and mandible were statistically significant (chi-squared test, *p* = 0.005).

Data were distributed normally for primary stability in the maxilla and mandible (Shapiro–Wilk test, *p* ≥ 0.132) and for secondary stability in the maxilla and mandible (*p* ≥ 0.145). RFA returned a mean ISQ of 71.2 ± 10.6 ISQ units for primary stability (Figure 3a,b) and 73.7 ± 13.3 ISQ units for secondary stability (Figure 4a,b) (*t*-test for paired samples, *p* = 0.213). There were no significant differences between the maxilla and mandible (*t*-test for independent samples, *p* ≥ 0.066).

### 3.4. Crestal Bone Changes

**Figure 5:**
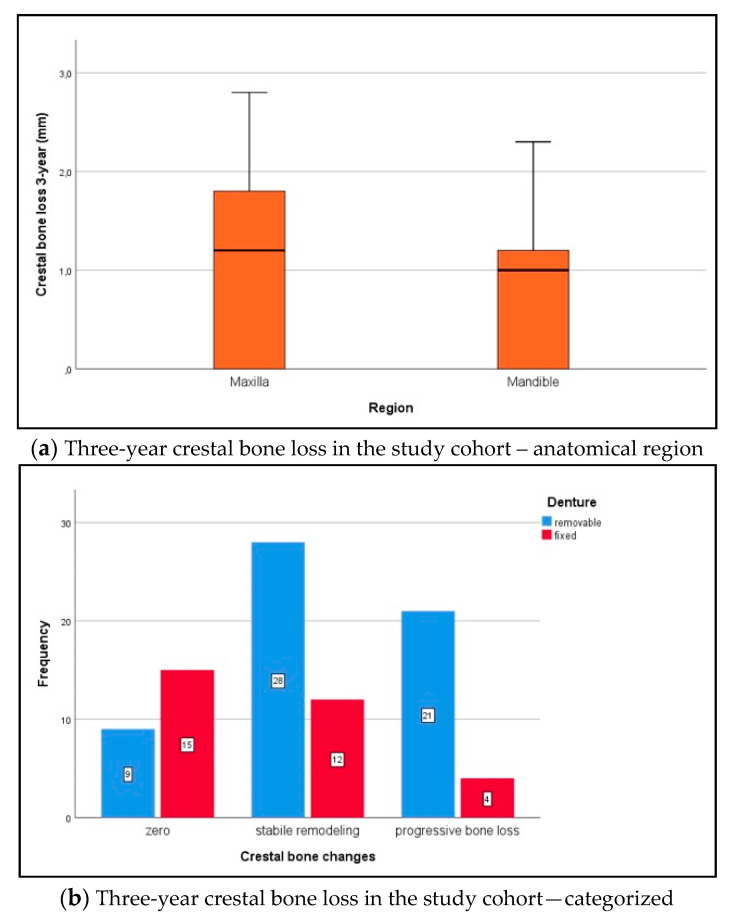
(**a**) Over the follow-up period, median crestal bone changes (mm) under implant loading were compared to the baseline: 1.2 (IQR 0–1.9) in the maxilla and 1.1 (IQR 0.1–1.2) in the mandible (Wilcoxon rank sum test, *p* = 0.304). (**b**) Crestal bone changes were categorized as “zero bone loss” = 0 mm, “stable bone remodeling” ≤ 1.2 mm, and “progressive bone loss” > 1.2 mm [32]. Changes in the fixed denture group were as follows: “zero bone loss” n = 15 (16.9%), “stable bone remodeling” n = 12 (13.5%), and “progressive bone loss” n = 4 (4.5%). Changes in the removable denture group were as follows: “zero bone loss” n = 9 (10.1%), “stable bone remodeling” n = 28 (31.5%), and “progressive bone loss” n = 21 (23.6%; chi-squared test, *p* = 0.002).

The 3-year crestal bone changes in the maxilla and mandible (Shapiro–Wilk test, *p* ≤ 0.013) were distributed non-normally. During the abovementioned follow-up period, median crestal bone changes (mm) under implant loading were compared to the baseline, as follows: in the first year, 1.0 (IQR 0–1.3) in the maxilla and 1.0 (IQR 0.2–1.2) in the mandible (maxilla vs. mandible Wilcoxon rank sum test for independent samples, *p* = 0.508); in the second year, 1.1 (IQR 0–1.3) in the maxilla and 1.0 (IQR 0.1–1.2) in the mandible (maxilla vs. mandible *p* = 0.382); in the third year (Figure 5a), 1.2 (IQR 0–1.9) in the maxilla and 1.1 (IQR 0.1–1.2) in the mandible (maxilla vs. mandible p = 0.304). Accordingly, crestal bone changes at implant sites were assessed in the maxilla as “zero bone loss” n = 11 (12.4%), “stable bone remodeling” n = 18 (20.2%), and “progressive bone loss” n = 15 (18.0%). Crestal bone changes in the mandible were assessed as “zero bone loss” n = 13 (14.6%), “stable bone remodeling” n = 22 (24.7%), and “progressive bone loss” n = 9 (10.1%). Maxilla-versus-mandible differences were not statistically significant (chi-squared test, *p* = 0.392). Categorized 3-year crestal bone changes according to prosthetic rehabilitation are presented in Figure 5b.

**Figure 6:**
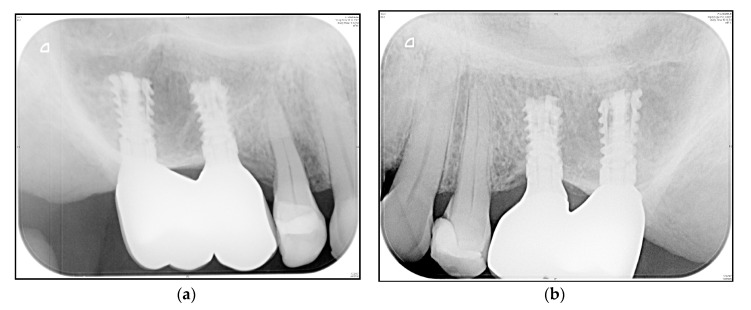
Posterior maxilla of a rehabilitated 62-year old woman at 1-year follow-up (Patient 11; Table 3). (**a**) Standard periapical radiogram implants i16 and i15; (**b**) Standard periapical radiogram implants i25 and i26.

**Figure 7:**
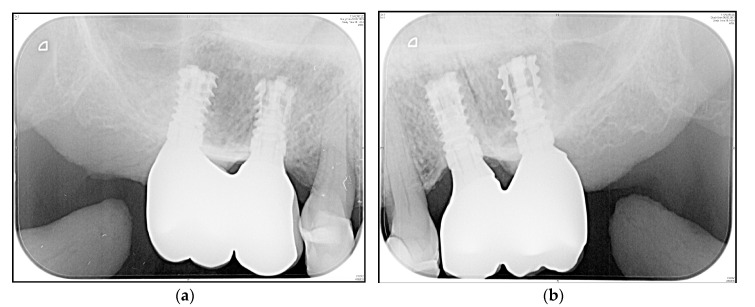
Posterior maxilla of a rehabilitated 62-year old woman at 3.5-year follow-up (Figure 6). (**a**) Standard periapical radiogram implants i16 and i15; (**b**) Standard periapical radiogram implants i25 and i26.

A representative “zero bone loss” case in a rehabilitated female patient is depicted in Figure 6a,b (1-year follow-up radiograms) and Figure 7a,b (3.5-year follow-up radiograms).

## 4. Discussion

Our study sought to evaluate a new expandable dental implant system under difficult, local bony conditions. Only a few reports in the literature have addressed expandable dental implants, and no other studies report on a comparable expandable short implant. The advantages and potential limitations of short and expandable implants are discussed in our earlier paper [24].

Recent literature has shown that short implants are increasingly accepted in the field of oral implantology [6,7,39,40]. The survival rate for short dental implants has increased from 80% to >90% over time [39]. This was also confirmed in two recent studies. For short dental implants that support single crowns and fixed bridges, especially in the mandible, a 2-year success rate of 97% [41] and 5-year outcome of 92.2% [7] were reported. Results from a 3-year randomized controlled trial indicated that short implants achieve similar outcomes, compared to longer implants that are inserted into augmented bone, and might be preferable compared to bone augmentation, especially in the posterior mandible [42]. According to another prospective 10-year cohort study, follow-up of single crowns supported by short implants in posterior regions and loaded after 6–7 weeks maintained long-term full function in 91.7% of cases, with low marginal bone loss (0.8 ± 0.7 mm) [43].

We achieved an overall implant success rate of 94.7% in the mandible and 83.6% in the maxilla for our elderly heterogeneous cohort (medical condition, indication category, implant site distribution, prosthodontic treatment), which is not fully comparable with results in the recent literature. The heterogeneity of the study cohort is therefore considered a weakness of the study design. We addressed this by presenting stratified results according to comorbidities and local conditions (anatomical region, bone quality, minor augmentations, prosthetic rehabilitation). There is a lack of directly comparable data from earlier clinical studies using this short implant design. In our study, very few implants were lost before loading and under loading (four vs. four, Table 3). The six patients that were affected presented compromised bone quality. This study returned a lower overall survival rate for implants in the maxilla than studies conducted by Slotte et al. (2015) [7] and Malmstrom et al. (2016) [41], which can be attributed to less optimal patients being included in our study. In a previous systematic review, 11 studies reported more implant failures before loading, while seven studies stated more implant failures under loading [39]. Furthermore, it is evident that there is a lower risk of complications when using short implants, compared to standard implants, in situations involving vertical bone loss that requires augmentation [2,4,5] or nerve lateralization [40].

Long-term success in implantology depends on a sufficient quantity and quality of peri-implant bone, as well as healthy mucosa. A wide range of modalities can be used to fully describe bone health, including biomarkers in serum and urine, imaging techniques, biomechanical testing, and histomorphometry [44]. Furthermore, studies on dental implant treatment present ambiguous bone tissue characteristics. In addition to the widely used Lekholm and Zarb classification, other authors describe bone quality using the Misch, Trisi, and Rao classification systems [45]. Structural jawbone quality relates to the amount of cortical and trabecular bone, while bone density relates to the amount of mineralization. The most precise methods of evaluating trabecular microstructure and bone density are multislice computed tomography (CT), cone beam CT, and micro-CT [46]. Triches et al. analyzed the relationship between insertion torque and the tactile, visual, and gray-value measures of bone quality with short implants, concluding that assessment methods are consistently related [47].

### 4.1. Oral Health-Related Quality of Life

In a representative normal population, OHIP scores typically tend to increase with age, along with a decreasing number of natural teeth, with scores ranging between 10 and 34 points [48,49,50]. According to a national population-based study, reference OHIP-14 values among 90% of subjects without dentures yielded ≤11 points, ≤17 points for subjects with a removable partial denture, and ≤25 points for subjects with a complete denture [27]. Based on the data collected in a national survey, John et al. (2004) concluded that specific OHIP scores can also provide a frame of reference for specific oral conditions, when OHRQOL is measured. Therefore, we believe that such evidence can be used as a model of comparison for our results. In the present study, the initial median OHIP score was 23 (IQR 9–25.5), which corresponds to subjects with removable dentures. The median decrease in OHIP cumulative scores was about 21 points to 2 (IQR 0–5), which corresponds to subjects without dentures. Our findings indicate a relevant increase in OHRQOL, suggesting successful patient-based oral rehabilitation, which aligns with results reported by John et al. [27] and Reißmann et al. [49].

### 4.2. Implant Stability under Difficult Conditions

Several investigators have analyzed the preferred indications of short dental implants in posterior mandibles and maxillae, and they have also outlined its cost efficiency compared to additional vertical augmentations. Our study applied a short implant in both jaws, with almost all possible indication categories represented, which confirms the broad versatility of the implant (Table 3).

Design modification (apical expansion) is aimed at gaining stability under difficult conditions, increasing bone-to-implant contact [50], and reducing the healing period [39,51,52,53,54]. It also impacts long-term crestal bone stability.

Earlier biomechanical finite element studies have confirmed that apical expansion results in a favorable stress reduction of almost 10% in the crestal bone [55]. We can assume that, in addition to the microthread and platform-switching concept [56], peri-implant crestal bone strain could be reduced by apical expansion.

With regards to RFA, the values obtained were related to bone quality and quantity, as well as the exposed implant height above the alveolar crest, which depends on the type of implant and insertion technique [57]. Our results (mean primary stability of 66.1 ISQ units in the maxilla and 75.9 ISQ units in the mandible; mean secondary stability of 68.2 ISQ units in the maxilla and 80.1 ISQ units in the mandible) were comparable to those obtained with standard-length implants. Becker et al. actually collected similar data (standard-length implants): primary stability of 72.1 ISQ units and secondary stability of 72.6 ISQ units [58]. These values are marginally lower than those of short implants that are only inserted into the posterior mandible (79.0 ISQ units) [9]. Other authors measured 68.2 ISQ units in the posterior maxilla (6 mm implants) [42]. Overall, the special design of the current study yielded reliably high stability values, compared to standard implant dimensions [30].

Although lateral augmentations are only performed in a few patients, they cannot be avoided in cases of a narrow alveolar ridge. Nevertheless, benefit–risk evaluation reveals that patients benefit from employed procedures without undergoing extensive additional vertical augmentations. In the present study, the apical implant design was shown to influence implant stability and bone-to-implant contact, which reinforces the findings of Romanos et al. [16] and Gehrke et al. [59]. The expansion procedure presents an additional bicortical anchorage in the oro-vestibular direction (pyramid shape of the alveolar process), which is understood to optimize load resistance [60]. In hard bone, manufacturers’ recommendations should be strongly considered. We found only one study reporting trans-crestal sinus lifting (average residual bone height of 4.7, using platelet concentrates) in association with 41 short implants [61], which is comparable to the case presented in Figure 6a and Figure 7b. The authors concluded that a stable augmented height gain of 4.2 mm was found after a 3-year follow-up.

Examples of alternative methods that aim to achieve a high level of biomechanical stability are osseodensification [62] and special thread designs [16,63]. For the posterior mandible, a comparative study of immediate loading (single crowns) on short implants and conventional dental implants reported comparable 1-year results for implant survival, marginal bone level, and ISQ values [64].

### 4.3. Peri-Implant Crestal Bone Loss

In the current study, crestal bone changes under loading in the first year were lower than in the second year, which did not differ from those obtained in the third year. Moreover, there were marginal differences between the maxilla and mandible in the first year, which only partially agrees with previous observations that have been reported in the literature [4,56].

Microbiological conditions influence the maintenance of peri-implant bone and play a critical role already during the osseointegration period. Clinical and microbiological analyses demonstrated an increased severity of marginal bone loss around non-submerged implants in relation to the salivary microbiome, particularly for participants with increased proportions of periodontal pathogenic species [65]. In a recent review of the metagenomics and culturomics of a peri-implant microbiome, Martellacci et al. (2019) asserted that teeth and implants do not appear to share the same microbiome, which is more diverse in healthy teeth [66]. Peri-implant and subgingival microbiota can more precisely be characterized by adopting culturomics [67]. The authors isolated a large amount of “uncultivable” species, and of 48 species, only 30 had been previously identified by metagenomics.

Another factor that affects peri-implant bone conditions is abutment morphology [68,69]. Furthermore, specific abutment surface characteristics (anodization) seem to be associated with a better soft-tissue outcome (greater height of keratinized mucosa) [70].

In addition to microbiological conditions, there are several biomechanical aspects that influence maintenance of peri-implant crestal bone. Conical and parallel surfaces of the implant-abutment connection (internal hexagon of the employed implant) provide rotational stability and combine the advantages of reduced microgaps and micromovement [71]. The implant shoulder exhibits microthreads and the platform-switching concept to reduce peri-implant bone strain [56,60]. The innovative macro-design enables apical expansion, which thus increases stability, in addition to enabling bone-to-implant contact. Another essential factor is the thickness of the implant shoulder [71], which may be a weak point in the design of a short implant, due to potential elastic deformity under extra-axial loading. This feature may be the reason for non-inflammatory peri-implant crestal bone loss. We addressed this aspect by splinting adjacent implants wherever possible [72]. Brenner et al. [18] and Pommer et al. [53] have suggested that prosthodontic factors should be considered to avoid screw loosening, component fracture, loss of marginal bone, or even loss of osseointegration. Different attachment systems (e.g., locator vs. ball attachment) and their varying heights clearly impact stress distribution at the implant neck [73]. Another essential factor is the peri-implant soft tissue in severely resorbed alveolar bone. Due to a loss of fixed/keratinized mucosa with progredient vertical bone atrophy, soft tissue conditioning must be meticulously addressed [74,75,76]. Therefore, based on the authors’ experience, implantological treatment using short implants requires adequate surgical skills.

Comparable studies have reported a crestal bone loss of 0.5–0.6 mm at 24 months [41]. Other authors reported a mean loss of 0.57 mm, 0.55 mm, and 0.53 mm in the mandible (without significant change after 1 year) [7]. Conversely, randomized controlled trials demonstrated peri-implant marginal bone loss of 0.7 mm at 1 year after loading [77] and 1.1 mm [4] in the maxilla, which is the same median value as that measured in the present heterogeneous study cohort over the 3-year period (at first year: 1.0 mm [IQR 0–1.3] in the maxilla and 1.0 mm [IQR 0.2–1.2] in the mandible; at second year: 1.1 mm [IQR 0–1.3] and 1.0 mm [IQR 0.1–1.2], respectively; at third year: 1.2 mm [IQR 0–1.9] and 1.1 mm [IQR 0.1–1.2], respectively). Long-term results from a randomized controlled trial, at 8 years after loading, showed that short implant patients lost significantly less crestal bone in the posterior mandible (an average of 1.6 mm vs. 2.5 mm in the augmented group) [78].

In comparison with conventional hollow-screw implants, concerns regarding expandable implants include the presence of gaps down to the apical region (potential microleakage that is comparable to distractible implants and endodontically treated teeth) and potential technical complications (the possibility that the implant cannot resist the load transmission). Both aspects were examined in vitro, which means the implant was submitted to microbiological and mechanical tests. Over the 3-year follow-up period, and in accordance with the study’s initial results, we did not observe any inflammatory signs in the apical region, either clinically or radiologically (Figure 6a,b and Figure 7a,b), or technical complications in terms of fracture.

### 4.4. Limitations of the Study and External Validity

The main limits of the study are its single-arm study design, and thus the inherent lack of a control group, and the heterogeneous baseline characteristics of the study cohort. The authors regard systemic comorbidities and local conditions as confounding factors. Therefore, generalization of the present findings to other settings should be performed with caution. Nevertheless, in terms of validating the initial outcomes, this larger trial sought to reevaluate the long-term safety of a specific short dental implant system. Due to their broad indication categories, the results of this study should be replicable in other settings with comparable patient characteristics and practitioner experience. In terms of future research, a randomized clinical trial should compare the presented macro-design with other short implants (e.g., a root-shaped, progressive thread design) in order to reduce the risk of confounding.

## 5. Conclusions

Within the limitations of a single-arm study and the mean 3-year follow-up period, the results demonstrate a reliable improvement in functional oral rehabilitation, especially for elderly patients whose general and local conditions make implantation difficult. The status of the alveolar process atrophy, the need for minor augmentation procedures, and the type of prosthetic rehabilitation (removable vs. fixed) significantly influenced the implant survival rate. Measures of OHRQOL were considerably enhanced. The tested implant was useful in terms of all bone qualities, as it exhibited high initial and secondary biomechanical stability in the maxilla and mandible. Median 3-year crestal bone changes under implant loading demonstrated maintenance of the peri-implant alveolar bone (stabile bone remodeling) in both jaws.

## Figures and Tables

**Table 1:** Inclusion and exclusion criteria.

Inclusion Criteria	Exclusion Criteria
Adult patients	Comorbidity ASA score > III
Partially or totally edentulous	Pregnancy, bruxism
Alveolar process atrophy (Cawood and Howell category III–IV)	Smoking ≥10 cigarettes/d
Minimum alveolar bone height of 7–9 mm for placement of short implants (5–7 mm length)	Patients with a significant risk of developing osteo(radio)necrosis of the jaw (radiotherapy ≥50 Gy, intravenous bisphosphonate therapy)
Patients that were not willing to accept vertical alveolar bone augmentation	Neurological and psychiatric comorbidities likely to influence the course of treatment
First implantological treatment	Untreated or poorly controlled diabetes mellitus, immunosuppression
Comorbidity ASA score I–III	Highly atrophic jaws that required vertical augmentation (Cawood and Howell category > IV)

ASA score: Preoperative medical status classification according to the American Society of Anesthesiologists.

**Table 2:** Surgical treatment protocol.

Surgical Protocol	Bone Quality			
	***D1*** (large homogenous cortical bone, little trabecular bone)	***D2*** (thick cortical bone, dense trabecular bone)	***D3*** (thin cortical bone, dense trabecular bone)	***D4*** (thin cortical bone, sparse trabecular bone)
Drilling protocol (splint)	last drill	last drill	second-to-last drill	second-to-last drill (cortical bone only)
2. Condensing preparation (osteotome technique)	-	-	-	analog to last drill
3. Implant insertion (maximum torque ≤40Ncm) 4. Expansion (maximum torque ≤40Ncm) 5. Measurement of primary stability (resonance frequency analysis), primary wound closure 6. Postoperative digital periapical radiogram 7. Re-entry after submerged healing (mandible: 3 months, maxilla: 6 months), measurement of secondary stability (resonance frequency analysis and insertion of healing abutments) 8. Postoperative digital periapical radiogram				

**Table 3:** Summary of the study cohort.

Patient * (Gender)	Age (Years)	ASA Score/ Systemic Disease	Surgery (Year)	Oral Disease/ Smoking	Implant Position (FDI; Σ)	Indication Category **	Bone Quality (Lekholm and Zarb)	Alveolar Process Atrophy (Cawood and Howell)	Prosthetic Treatment	Implant Failure	Follow-up Period (Months)
1. ^#^ (female)	80	2/ Diabetes mellitus, bronchitis, arterial hypertension	2014	None/ no	*Maxilla* **15**, 13, 11, 21, 25 (**Σ = 5**)	IIa	D4	IV	*Telescope*	*1 (before loading) ^##^*	64
2. ^#^ (female)	64	1/ Osteoporosis	2014	None/ no	*Maxilla* 14, 12, 22, 24 (**Σ = 4**)	IIIa	D4	IV	*Jaw bar*	None	61
3. ^#^ (male)	53	2/ Cured squamous-cell carcinoma (floor of the mouth)	2014	History of marginal periodontitis/ yes	*Maxilla* **16**, **14**, 12 (**Σ = 3**)	IIb	D3	IV	*Ball attachment*	*2 (under loading) ^##^*	60
4. (male)	67	1	2015	None/ no	*Maxilla* 17(2x, **17**), 13, **21**, 23, 27 (2x, **27**) (**Σ = 7**)	IIIa	D3-D4	IV	*Jaw bar*	*3 (before loading) ^##^*	55
5. (female)	62	1	2015	History of mid-facial trauma/ no	*Maxilla* 16, 14, 12, 22, 24, 26 (**Σ = 6**)	IIIa	D4	IV	*Jaw bar*	None	52
6. (male)	54	2/ Cured squamous-cell carcinoma (tongue)	2016	Chronic mucositis/ yes	*Maxilla* 14, 12, 22, 24 (**Σ = 4**)	IIIa	D3	IV	*Ball attachment*	None	40
7. (male)	50	1	2017	History of marginal periodontitis/ yes	*Maxilla* 15, 13, 23, 25 (**Σ = 4**)	IIIa	D3	III	*Ball attachment*	None	32
8. ^#^ (female)	44	1	2014	None/ no	*Maxilla* 16, 15, 14 (**Σ = 3**)	IIa	D3	III	Bridge	None	60
9. (male)	76	1	2015	None/ no	*Maxilla* 16, 15, 14 (**Σ = 3**)	IIb	D3	III	Bridge	None	56
10. (male)	61	1	2015	None/ no	*Maxilla* 24, 25 (**Σ = 2**)	IIb	D2	III	Bridge	None	51
11. (female)	62	1	2015	History of marginal periodontitis/ no	*Maxilla* 16, 15, 25, 26 (**Σ = 4**)	IIb	D4	IV	Bridge	None	48
12. (male)	57	1	2015	History of marginal periodontitis/ no	*Maxilla* 26, 27 (**Σ = 2**)	IIb	D3	III	Bridge	None	47
13. (male)	53	1	2016	None/ no	*Maxilla* 25, 26 (**Σ = 2**)	IIb	D3	III	Bridge	None	46
14. (female)	58	2/ Cured HCV infection	2016	History of marginal periodontitis/ yes	*Maxilla* 16, 14, 25, 26 (**Σ = 4**)	IIb	D3-D4	III	Bridge	None	41
15. ^#^ (female)	65	2/ Chronic bronchitis, arterial hypertension	2014	None/ no	*Mandible* 34, **32**, 42, 44 (**Σ = 4**)	IIIb	D1	IV	*Ball attachment*	*1 (under loading) ^##^*	61
16. ^#^ (female)	72	2/ Osteoarthrosis	2015	History of marginal periodontitis/ no	*Mandible* 42, 44, 46 (**Σ = 3**)	IIb	D2	IV	*Ball attachment*	None	50
17. (female)	74	3/ Congestive heart failure, arterial hypertension, sicca syndrome	2016	None/ no	*Mandible* 34, 32, 42, 44 (**Σ = 4**)	IIIb	D3	IV	*Ball attachment*	None	43
18. (male)	69	3/ Cured squamous-cell cancer (oropharynx)	2016	Chronic mucositis/ yes	*Mandible* 34, 32, 42, 44 (**Σ = 4**)	IIIb	D2	IV	*Jaw bar*	None	41
19. (female)	63	2/ Hypothyroidism	2016	None/ no	*Mandible* 34, 32, 42, 44 (**Σ = 4**)	IIIb	D3	IV	*Jaw bar*	None	41
20. (female)	66	2/ Osteoporosis	2016	History of marginal periodontitis/ no	*Mandible* 35, 45 (**Σ = 2**)	IIb	D3	IV	*Ball attachment*	None	40
21. (male)	76	3/ Arteriosclerosis, hemiplegia	2016	None/ yes	*Mandible* 34, 32, 42, 44 (**Σ = 4**)	IIIb	D2	IV	*Jaw bar*	None	37
22. (male)	59	2/ Venous thrombosis	2016	None/ no	*Mandible* 34, 32, **42**, 44 (**Σ = 4**)	IIIb	D2	IV	*Ball attachment*	*1 (under loading) ^##^*	37
23. (female)	80	3/ Coronary arteriosclerosis, congestive heart failure	2017	None/ no	*Mandible* 34, 32, 42, 44 (**Σ = 4**)	IIIb	D2	IV	*Ball attachment*	None	32
24. (female)	83	3/ Cured squamous-cell carcinoma (maxilla)	2017	Leukoplakia/ no	*Mandible* 34, 32, 42, 44 (**Σ = 4**)	IIIb	D2	IV	*Ball attachment*	None	32
25. (female)	55	1	2018	None/ no	*Mandible* 35, 31/41, 45 (**Σ = 3**)	IIIb	D2	IV	*Ball attachment*	None	21
26. ^#^ (male)	76	1	2014	None/ no	*Mandible* 35, 36, 37 (**Σ = 3**)	IIb	D1	III	Bridge	None	60
27. ^#^ (female)	52	1	2015	History of marginal periodontitis/ no	*Mandible* 35, 36, 37 (**Σ = 3**)	IIb	D2	III	Bridge	None	56
28. ^#^ (female)	59	1	2015	None/ no	*Mandible* 35, 36 (**Σ = 2**)	IIb	D2	IV	Bridge	None	51
29. (female)	59	2/ Gastric ulcer	2016	None/ no	*Mandible* 47 (**Σ = 1**)	Ib	D2	III	Crown	None	44
30. (female)	48	1	2016	None/ yes	*Mandible* 35, 36 (**Σ = 2**)	IIb	D2	III	Bridge	None	37

ASA score: Physical status classification according to the American Society of Anesthesiologists. FDI: Fédération Dentaire Internationale. HCV: Hepatitis C virus. Σ: Total implants per patient. ^#^: Short-term results of these nine patients were previously published [24]; two additional implants (Patients 3 and 5) were lost to long-term follow-up. *: Over the follow-up period, four patients dropped out (non-compliance: three patients, malignancy: one patient; total: 13 implants). These patients are not included in this table. **: Indication categories related to the recommended implant amount, according to the German consensus conference [32]; modified in Patients 2, 9, and 23. ^##^: In total, five patients experienced implant loss: four patients with alveolar process atrophy (Cawood and Howell category IV [20]) and one patient (Patient 5) with a history of oral squamous-cell carcinoma. Implant number (strikethrough): Lost implant. Implant number (underlined): Additional bone augmentation at n = 23 implant sites (lateral augmentation n = 4, bone spreading n = 12, internal sinus lift n = 7).

**Table 4:** Summary of all dimensions of preoperative and postoperative OHIP-14 scores.

OHIP-14 Dimension	Variables	Baseline Median (IQR)	Post-Rehabilitation Median (IQR)	Statistics (Wilcoxon Signed Rank Test for Paired Samples)
*Functional limitation*	Have you had trouble pronouncing any words because of problems with your teeth, mouth, or dentures?	1 (0–2.5)	0 (0–0.5)	*p* = 0.059
	Have you felt that your sense of taste has worsened because of…?	0 (0–1.5)	0 (0–0)	*p* = 0.194
*Physical pain*	Have you experienced painful aching in your mouth?	2 (0–2)	0 (0–1.5)	*p* = 0.168
	Have you found it uncomfortable to eat any foods?	3 (1–3.5)	1 (0–1.5)	*p* = 0.039 *
*Psychological discomfort*	Have you been self-conscious about…?	2 (0.5–3)	0 (0–0)	*p* = 0.026 *
	Have you felt tense?	2 (0–2.5)	0 (0–1)	*p* = 0.031 *
*Physical disability*	Has your diet been unsatisfactory?	2 (0.5–3.5)	0 (0–2)	*p* = 0.071
	Have you had to interrupt meals?	2 (0.5–2.5)	0 (0–0)	*p* = 0.027 *
*Psychological disability*	Have you found it difficult to relax?	1 (0.5–2.5)	0 (0–1)	*p* = 0.071
	Have you been slightly embarrassed?	1 (0.5–2)	0 (0–0)	*p* = 0.015 *
*Social disability*	Have you been slightly irritable around other people?	1 (0.5–2)	0 (0–0)	*p* = 0.023 *
	Have you found it difficult to perform your usual jobs?	1 (0–2)	0 (0–0.5)	*p* = 0.068
*Handicap*	Have you felt that life in general was less satisfying?	2 (2–3)	0 (0–2)	*p* = 0.041 *
	Have you been totally unable to function?	0 (0–1)	0 (0–0)	*p* = 0.157

*: Statistically significant differences between **median** (baseline) and **median** (after prosthetic rehabilitation). IQR: Interquartile range.

## Supplementary Materials

The following are available online at https://www.mdpi.com/1010-660X/56/7/333/s1. The STROBE checklist and relevant raw data for pseudonymized patients are attached to a SPSS-databank.
